# Acute hepatotoxicity of intravenous amiodarone in a Becker muscular dystrophy patient with decompensated heart failing and *ABCB4* gene mutation: as assessed for causality using the updated RUCAM

**DOI:** 10.1186/s13019-024-02869-7

**Published:** 2024-07-23

**Authors:** Hui Shi, Ruizhen Chen, Minghui Li, Junbo Ge

**Affiliations:** 1 Department of Cardiology, Zhongshan Hospital, Fudan University, Shanghai, China; 2 Department of Cardiology, Shanghai Institute of Cardiovascular Diseases, Zhongshan Hospital, Shanghai Medical College of Fudan University, 1069 Xietu Road, Shanghai, 200032 China; 3 National Clinical Research Center for Interventional Medicine, Shanghai, China

**Keywords:** Becker muscular dystrophy, Amiodarone, *ABCB4*, Acute hepatotoxicity, Case report, RUCAM

## Abstract

**Background:**

Cardiac dysfunction, including arrhythmias, may be one of the main clinical manifestations of Becker muscular dystrophy (BMD). Amiodarone is widely used to treat arrhythmia. However, multi-systemic toxicity caused by amiodarone, especially hepatotoxicity, should not be neglected. Here, we introduce a novel case of multi-systemic amiodarone toxicity involving the liver, renal and coagulation in BDM patient with *ABCB4* gene mutation.

**Case presentation:**

We present a case of a 16-year-old boy admitted with heart failure and atrial fibrillation (AF). He was diagnosed with Becker muscular dystrophy (BMD) and gene testing showed comorbid mutations in gene *DMD*, *ABCB4* and *DSC2*. Amiodarone was prescribed to control the paroxysmal atrial fibrillation intravenously. However, his liver enzyme levels were sharply elevated, along with cardiac shock, renal failure and coagulation disorders. After bedside continuous renal replacement therapy, the patient’s liver function and clinical status rehabilitated.

**Conclusions:**

*ABCB4* gene mutation might be involved in amiodarone-induced hepatotoxicity. Studies in a cohort might help to prove this hypothesis in the future.

## Background

Becker muscular dystrophy (BMD), is an X-linked neuromuscular disease caused by dystrophin gene mutation which results in a partial defect of dystrophin, whereas a complete loss of the protein causes the Duchenne muscular dystrophy(DMD) [1]. Cardiac dysfunction, including arrhythmias and dilated cardiomyopathy (DCM), may be one of the main clinical manifestations of both DMD and BMD, and sometimes appears to be the only initial manifestation without skeletal muscle weakness in BMD. Actually, cardiovascular complications are a leading cause of mortality in BMD [2].

ATP-binding cassette (ABC) subfamily B member 4 (ABCB4), encoded by *ABCB4* gene, is involved in protecting hepatobiliary system from deleterious detergent. ABCB4 aberrations may be involved in some cases of drug-induced liver injury (DILI) like oral-contraceptives, psychotropic drugs, selected chemotherapy drugs, statins, and antibiotics [3, 4]. So far as we know, no report about the role of ABCB4 deficiency in amiodarone-induced liver injury has been reported before.

We present here a BMD patient complicated with *ABCB4* gene mutation who experienced acute hepatotoxicity after intravenous (IV) amiodarone administration. This report follows the DILI Study Group, Chinese Society of Hepatology (CSH), Chinese Medical Association (CMA) and CSH guidelines for the diagnosis and treatment of drug-induced liver injury [5].

## Case presentation

The patient was a 16 year-old boy who was referred to our hospital in July 2022 because of severe heart failure.

At age 13, he was diagnosed Becker muscular dystrophy carrying with the causative mutation in gene *DMD (chrX:32,841,413–32,862,977, Hemizygous mutation)*, and gene testing showed the comorbid mutations of *ABCB4(chr7:87041219, heterozygous mutation)* and *DSC2(chr18:28659938, heterozygous mutation)*. He had a history of mild breath shortness and chest distress on exertion and received treatment for cardiac dysfunction; however, heart function became worse and he was referred to our hospital for the first time. Physical examination at his first referral showed a height of 173 cm, body weight of 78 kg, blood pressure of 110/90 mmHg, heart rate of 76 beats/minute regularly, respiratory rate of 20 times/minute, body temperature of 36.5 °C, and SpO2 of 100% under room air. There was no jugular vein dilatation in the neck. His lung and cardiac auscultation sounds were normal. His abdomen was flat. There was slight edema in both instep and ankle. The patient did not show wing-like shoulder blades or lordosis. Pseudohypertrophy of the bilateral gastrocnemius muscle was observed. Gower’s sign was negative. Laboratory test showed that N-terminal prohormone of brain natriuretic pee (NT-proBNP) level was 471.55pg/ml, myohemoglobin was 272.61ng/ml, creatine kinase (CK) was 5,493 U/L, creatine kinase-MB (CK-MB) was 115 U/L, creatine kinase-MM (CK-MM) was 5,378U/L. Alanine aminotransferase (ALT) was 138 U/L, aspartate aminotransferase (AST) was 92 U/L, lactate dehydrogenase (LDH) was 393U/L. The transthoracic echocardiography showed a 19% left ventricular ejection fraction (LVEF), dilation of left atrium, left ventricle and right atrium, mild mitral valve regurgitation, severe LV systolic dysfunction, decreased whole heart diastolic function. Leg MRI revealed that muscular dystrophy changes in bilateral thighs. Medication treatments were started including sacubitril-valsartan, carvedilol, ivabradine, tolvaptan, trimetazidine and adjusted according to the heart failure treatment guidelines in the coming 3 years’ of follow-up. Follow-up echocardiographic LVEF value fluctuated at 30–35%.

On the first day of this referral, the patient suffered deteriorating chest distress, palpitation, and shortness of breath at rest indicating progressive heart failure. He was referred to our hospital at 9:00 p.m. On physical examination, blood pressure of 106/63mmHg, heart rate of 120 beats/minute irregularly, respiratory rate of 20 times/minute, body temperature of 37℃, and SpO_2_ of 97% on room air. A chest examination revealed moist crackles in both lungs. His heart sounds were intact but irregular, and no heart murmur was observed. His muscle strength was 5/5. Laboratory tests showed the following results: white blood cell count, 10.56 × 10^9^/L with neutrophils 60.5%; hemoglobin, 144 g/L; platelet count, 177 × 10^9^/L. Coagulation function was sightly abnormal, as follows: Prothrombin time (PT), 14.8 s; activated partial thromboplastin time (APTT), 27.1 s; D-Dimer, 0.27 mg/L. Biochemical examination showed potassium of 3.9 mmol/L, creatinine of 67µmol/L. His total bilirubin level was normal, but his liver enzyme levels were slightly elevated, as follows: ALT, 109 U/L; AST, 87 U/L; LDH, 376U/L. NT-proBNP was markedly elevated to 9,171pg/ml. cTnT level was 0.107ng/ml. The electrolyte levels were normal. Electrocardiogram showed atrial fibrillation with rapid ventricular rate and intraventricular heart block.

At 10:00 p.m. on the first day, chest distress was aggravating. 150 mg of amiodarone through intravenous injection failed cardioversion. Ventricular rate was 120 bpm. Then, 450 mg of amiodarone was used for rate and rhythm control through continuous intravenous dripping (6ug/Kg/min). At the same time, we provide continuous infusion of recombinant human brain natriuretic peptide to improve clinical heart function. ECG monitor showed atrial fibrillation with ventricular rate of 90 bpm.

At 1:00 pm on the second day, the patient became pale, sweating and orthopnea. The patients’ blood pressure dropped to 75/50 mmHg, heart rate was 74 bpm. Therefore, amiodarone and recombinant human brain natriuretic peptide was discontinued and intravenous infusion of dopamine and dobutamine was administrated. All oral medications were stopped at the same time. ECG showed a normal sinus rhythm of 84 bpm and second-degree type II atrioventricular block. Emergency bedside echocardiography showed that dilatation of whole heart with reduced systolic function of left and right ventricle (LVEF 25%), mild pulmonary artery hypertension, infinitesimal pericardial effusion. Physical examination showed blood pressure of 89/60mmHg, heart rate of 74 bpm regularly and SpO2 of 99% under 3 L/minute oxygen. Skin mottling was visible on both lower limbs. The urine volume on the second day was 1,000 ml.

On the third day, abdominal ultrasound revealed gallstones and cholecystitis. Meropenem (0.5 g q12h ivgtt) was used empirically to control infection. The urine volume decreased to 400 ml.

On the 4th day, the patient complained of relief of chest distress. Laboratory tests showed the following results: ALT, 7,391 U/L; AST, 5,671 U/L; LDH, 8,089U/L; CK, 2,875pg/ml, CK-MM, 28,522U/L; CK-MB, 229U/L; urea nitrogen, 29.8mmol/L; uric acid, 859µmol/L; creatinine, 305 µmol/L; eGRF, < 30 ml/min/1.73m^2^; platelet,104 × 10^9^/L; PTs, 29.3 s; APTT, 29.9 s; Fibrinogen, 163 mg/dl; D-Dimer > 40 mg/L. He was referred to ICU for further treatment.

We provided bedside continuous renal replacement therapy. Administrations of isoglycyrrhizinate, reduced glutathione and polyene phosphatidylcholine were given to protect liver function. Dopamine and dobutamine (1.3ug/kg/min) was administrated to sustain blood pressure and strengthen heart contraction. Laboratory examinations were conducted to monitor patient’s related indexes. Coagulation function: PTs was 22 s. D-Dimer was higher than 40 mg/L. Liver function were as follows: total bilirubin level of 75.5µmol/L, ALT of 5,060 U/L, AST of 1,952 U/L, LDH of 8,089U/L. Renal function: blood urea nitrogen of 23.8mmol/L, uric acid of 604 µmol/L, creatinine of 322 µmol/L, and eGRF 23 ml/min/1.73m^2^. NT-proBNP was 5701 pg/ml, CK was 11476U/L, CK-MM 11308U/L, CK-MB 168U/L. ECG showed a normal sinus rhythm of 62 bpm, intraventricular heart block, left atrial hypertrophy and left axis deviation.

At 10:00 pm on the 5th day, dopamine was discontinued. Blood pressure fluctuated 90–112/50-70mmHg. We administrated oral sacubitril-valsartan 25 mg bid and trimetazidine 35 mg bid on July 10th. Ivabradine (5 mg bid p.o.) and tolvaptan (10 m qd p.o.) were administrated on the 10th day due to stable hemodynamics, improved renal function and increased daily urine volume. Dobutamine was stopped on the 11th day. Low molecular weight heparin was started on the 12th day.

**Fig. 1:**
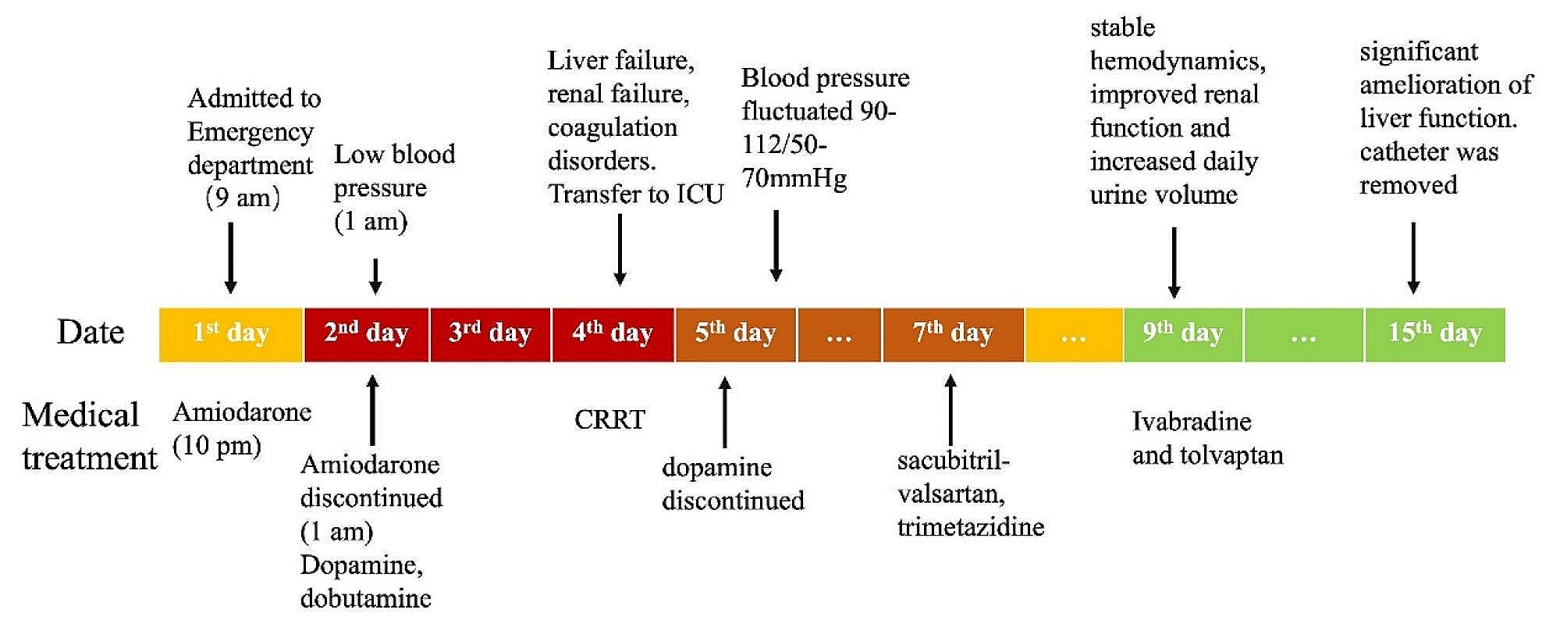
The timeline with episodes of care and medical treatment for the patient

**Fig. 2:**
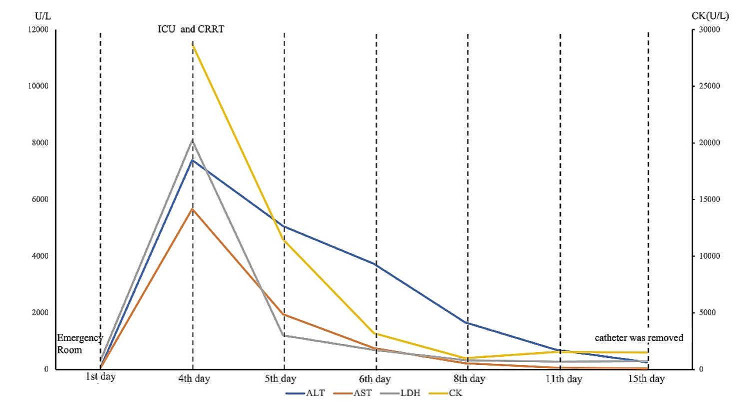
The timeline with dynamic alterations of ALT, AST, LDH and CK

On the 15th day, reexamination of laboratory examinations suggested significant amelioration of liver function. Alanine aminotransferase was down to 255 U/L, aspartate aminotransferase to 35U/L. The daily amount of transfusion was 2,106 ml with daily urinary output of 1,125 ml. Therefore, polyene phosphatidylcholine was stopped. Hemodialysis catheters were removed. A timeline with relevant data from the episode of care was showed in Figs. 1 and 2.

## Discussion and conclusions

Becker muscular dystrophy (BMD) is an allelic variant of Duchenne muscular dystrophy (DMD) and occurs at one-tenth the frequency of DMD [6]. Although the majority of BMD mutations are inherited, approximately 30% are due to *de novo* mutations, with no family history [7]. This case belongs to the latter spontaneous mutations. The family history is negative. The mutations are not found in his parents or grandparents. The origin of the mutations is unclear. It might be developed spontaneously during gametogenesis or following stages of embryonic development.

The manifestations of BMD may range from peripheral myopathy to severe congestive heart failure. However, cardiac involvement is a frequent clinical finding in BMD, and may progress and develop into DCM without symptoms [8] which has now emerged as the leading cause of death in BMD patients [9]. DCM typically has an onset in the mid-teen years and contributes to the death of patients with BMD.

The onset and course of cardiac involvement may be largely variable even in monozygotic triplets. In some BMD patients, cardiac dysfunction preceding the development of skeletal muscle weakness [10]. Myocardial damage may progress with the ability to perform vigorous physical exercise still present [11]. As presented in this report, the patient suffered more severe heart failure comparing with the mild myopathy clinical symptoms.

Arrhythmias, including atrial fibrillation, are also common cardiac involvement in patients with BMD. Amiodarone, a class III antiarrhythmic drug, is widely used for the treatment of both supraventricular and ventricular arrhythmias including atrial fibrillation.

In this case, the patient was admitted to our hospital with mild elevated liver enzymes, sightly abnormal coagulation function and normal renal function. Intravenous amiodarone (total amount was about 300 mg) was administrated due to the occurrence of atrial fibrillation with rapid ventricular rate. Consequentially, the patient’s liver function, renal function and coagulation function deteriorated sharply.

The etiologies of abnormal liver function usually consist of viral hepatitis, ischemia, autoimmune diseases, and medications [12]. This patient does not drink alcohol and is with no history of hepatitis. Abdominal ultrasonography and viral hepatitis serologies were conducted and hepatic vascular and virus diseases were excluded. No history of autoimmune disease or related evidence were found either.

Although poor perfusion (shock state) was an obvious source of liver damage, and it seems that we are dealing with a hypoxic liver injury that was perhaps more likely cause of liver injury, the involvement of IV amiodarone in causing acute hepatotoxicity could still not be excluded totally. First, the development of acute cardiogenic liver injury is also an exclusion diagnosis [13].The diagnosis is based on: (1) setting: cardiac, circulatory, or pulmonary failure; (2) aminotransferase levels, usually > 20 times the upper limit of normal; and (3) exclusion of other causes of liver damage. In this case, the patient was prescribed amiodarone, which is well-known for the hepatotoxicity, the probability of amiodarone-induced liver injury was still not excluded. Second, a RCRUM score [5] of 5 was achieved for this case, which indicated a possible drug-induced liver injury(DILI). Third, Van et al. found in 234 patients with acute heart failure that no substantial changes in liver function tests during hospitalization up to day 14 were observed, including 36 patients had in-hospital worsening heart failure within 5 days [14]. This is not coincided with what was observed in our case that both ALT and AST jumped to > 140ULN, comparing with about > 2ULN at baseline. Similarly, this patient has been hospitalized several times in our hospital for acute heart failure. During that time, his ALT levels fluctuated between 65 and 123 U/L. Therefore, it is hard to attribute such unusual upregulation of ALT and AST levels to sole hypoperfusion or cholecystitis, which also indicated the possible involvement of IV amiodarone in causing acute hepatotoxicity.

Additionally, a temporary atrioventricular conduction block (AVB) was observed shortly (2–3 h) after administration of IV amiodarone. Although ischemia of the conduction system due to poor perfusion (the patient was suffering a significant shock state) might be one of the reasons causing AVB, a probable transient high concentration of circulatory amiodarone might also be the causing agent, which indicated amiodarone might be a factor causing the deterioration. Finally, the patient’s condition became stable within a few days after amiodarone withdrawal. This retrospective evidence also supported that the acute hepatotoxicity might be caused by amiodarone.

However, the incidence of acute server hepatotoxicity of amiodarone is rare (about 1.1%) [15] after all and no report has been published in BMD patients’ treatment ever before so far as we know. Actually, it has been reported that chronic atrial fibrillation in BMD heart failure patients can be successfully treated with amiodarone [16]. Moreover, the acute hepatotoxicity of IV amiodarone usually occurs within 24 h after administration, and sometimes after more than 3 days [17]. But in this case, it happened 2 h after IV amiodarone administration. Why did so rare hepatotoxicity of IV amiodarone happen so quickly in a so little dosage?

The underlying mechanisms of hepatotoxicity of IV amiodarone are basically still unclear. Some reports postulated that the cause may not be amiodarone but the contents in amiodarone infusion including polysorbate 80 and a small amount of benzyl alcohol [18–20]. Some reports considered that dose may be the reason causing hyperacute hepatitis: daily dose of IV form up to 1500 mg seems to be more vulnerable to cause acute liver injury comparing with the daily dose of oral form of just 200 mg. But in this case, the dosage of IV amiodarone is rather little (total amount of about 300 mg). Are there any other underlying mechanisms leading to the fatal acute liver injury?

According to all the above, except hypoperfusion, a possible role of amiodarone in causing acute hepatotoxicity could not be excluded in this unique case. Amiodarone is a lipophilic drug with a pharmacokinetic profile characterized by large interpatient variability. And excretion of amiodarone occurs mainly in the bile [21].

In this case, the patient has a gene mutation in *ABCB4* in addition to the DMD. ABCB4 protein is involved in biliary phospholipid secretion, protecting hepatobiliary system from deleterious detergent and lithogenic properties of the bile [22]. Although the correlation of *ABCB4* mutation and amiodarone metabolism has not yet been reported, the well-known pathological significance of ABCB4 protein mutation provide the potentiality of amiodarone secretion retard, which we lack direct evidence to prove in this case report and may be studied in future research.

In the course of disease, the patient made several hospital visits due to cholecystitis. The ultrasound on 3rd day also suggested that the patient had cholecystitis. Therefore, it is rational to hypothesize that the severe liver function impairment may be related to the amiodaronen accumulation caused by the disabled excretion of amiodaronen from the bile.

Transaminases are usually used as a gold standard biomarker for diagnosis of the liver injury. However, elevated transaminase has limitations in the diagnosis of hepatocyte damage due to lack of liver specificity, especially in patients with muscle damage including BMD and DMD. In subjects with muscle damage, the increased transaminases released from damaged muscle tissue can effectively cover the ALT or AST activity originated from liver as a consequence of hepatocyte injury. While LDH is a liver specific enzyme which is not affected by muscle injury. Studies have reported that LDH in patients with BMD were equivalent to those in healthy subjects, whereas ALT levels were significantly higher in patients with BMD comparing with healthy people [23]. But no liver injury happened in those people with increased transaminase and normal LDH, indicating that LDH may be more specific to rule out possible liver injury. Therefore, many studies have pointed that LDH has great potential as an alternative to ALT for detection of liver injury in patients with BMD [24]. Therefore, in the subsequent treatment of BMD patients, especially in the terms of drug safety, the results of LDH can be comprehensively considered to monitor liver function, in order to avoid or identify the occurrence of drug-induced liver damage as early as possible.

In conclusion, IV amiodarone administration should be prescribed for BMD patients cautiously and it is suggested to closely monitor liver enzymes especially LDH. As *ABCB4* gene mutation might be one of the reasons causing acute hepatotoxicity, further studies might be helped to prove this hypothesis in the future.

## Data Availability

The data underlying this article will be shared upon reasonable request to the corresponding author.

## Abbreviations

BMD: Becker Muscular Dystrophy
DMD: Duchenne Muscular Dystrophy
AF: Atrial Fibrillation
CRRT: Continuous Renal Replacement Therapy
DCM: Dilated Cardiomyopathy (DCM)
ABCB4: ATP-Binding Cassette subfamily B member 4 (ABCB4)
ICU: Intensive Care Unit
IV: Intravenous
DILI: Drug-Induced Liver Injury
ALT: Alanine Aminotransferase
AST: Aspartate Aminotransferase
LDH: Lactate Dehydrogenase
LVEF: Left Ventricular Ejection Fraction
NT-proBNP: N-Terminal prohormone of Brain Natriuretic Pee
CK: Creatine Kinase
PT: Prothrombin Time
APTT: Activated Partial Thromboplastin Time
AVB: Atrioventricular Conduction Block
